# Online Condition Monitoring of a Rail Fastening System on High-Speed Railways Based on Wavelet Packet Analysis

**DOI:** 10.3390/s17020318

**Published:** 2017-02-08

**Authors:** Jiahong Wei, Chong Liu, Tongqun Ren, Haixia Liu, Wenjing Zhou

**Affiliations:** 1 Key Laboratory for Precision and Non-traditional Machining Technology of Ministry of Education, Dalian University of Technology, Dalian 116024, China; jh_wei@mail.dlut.edu.cn (J.W.); chongl@dlut.edu.cn (C.L.); 2 Key Laboratory for Micro/Nano Technology and System of Liaoning Province, Dalian University of Technology, Dalian 116024, China; liuhaixia0930@163.com; 3 The 45th Research Institute of CETC, Beijing 100176, China; apr_zhou@163.com

**Keywords:** rail fastening system, on-line monitoring, wavelet packet analysis, fasteners looseness

## Abstract

The rail fastening system is an important part of a high-speed railway track. It is always critical to the operational safety and comfort of railway vehicles. Therefore, the condition detection of the rail fastening system, looseness or absence, is an important task in railway maintenance. However, the vision-based method cannot identify the severity of rail fastener looseness. In this paper, the condition of rail fastening system is monitored based on an automatic and remote-sensing measurement system. Meanwhile, wavelet packet analysis is used to analyze the acceleration signals, based on which two damage indices are developed to locate the damage position and evaluate the severity of rail fasteners looseness, respectively. To verify the effectiveness of the proposed method, an experiment is performed on a high-speed railway experimental platform. The experimental results show that the proposed method is effective to assess the condition of the rail fastening system. The monitoring system significantly reduces the inspection time and increases the efficiency of maintenance management.

## 1. Introduction

**Figure 1:**
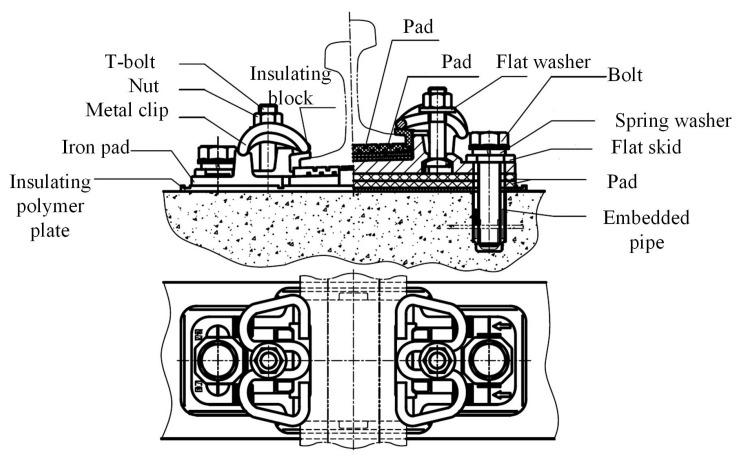
Assembly diagram of the WJ-7 fastening system.

The functions of a rail fastening system (Figure 1) are fixing the rail on the track slab and preventing the longitudinal and lateral movements of the rail. Meanwhile, through offering flexibility, the rail fastening system can also counteract the vertical wave movements of the track, from which would result in the reduction of the dynamic forces between wheel and rail [1,2]. The main components of a rail fastening system are metal clips, insulating polymer plate, and bolts. The components of a rail fastening system are vulnerable to fatigue damage due to wheel-rail interaction [3]. The reduction of frictional resistance between the T-bolt and nut causes the clamping force of a rail fastener to decrease. In this situation, the rail longitudinal resistance will not meet the design requirements. The loosening of the nut gives rise to intensifying the track irregularity, increasing the interaction between the rail and wheel, and may cause train derailment [4]. Therefore, the condition detection of rail fastening systems, for looseness or absence, urgently needs to be studied.

The current literature provides a few image-based methods for detecting rail fastener defects [5,6,7,8,9,10]. The methods mentioned above can detect the absence of fasteners effectively, but cannot identify the severity of rail fastener looseness. Structural damage identification based on vibration data has broad application prospects in structural health monitoring (SHM) [11,12]. Wang et al. [13] presented a method to identify the looseness of rail fastener, in which the self-power density method was used to analyze the vibration signals from four acceleration sensors installed on the railhead. The results revealed that the power spectral density was significantly decreased in the frequency of 2.95 kHz when the rail fastener loosened. Ren et al. [14] used quaternion-based three-channel joint transmissibility to detect the state of rail fasteners between two testing points. Their experimental results showed that the state indicator can identify the number of loosened fasteners. A method based on the combination of orthogonal empirical mode decomposition and the theory of time-frequency entropy was used to detect rail fastener state [15]. Their experimental results showed that this method can identify the severity of rail fasteners looseness to some extent, but the damage location is not considered. Additionally, the defects may not be spotted in time. Therefore, an automatic and remote-sensing measurement system used to monitor the condition of rail fasteners is always desirable.

Recently, the damage identification using structural dynamic responses based on time-frequency analysis has received growing attention in the field of railway structural health monitoring [14,15]. Oregui et al. [16] used a vehicle-borne monitoring system to detect and assess the tightness condition of bolts at rail joints based on wavelet analysis. The theoretical research [17] showed that a series of component signals can indicate the dynamic properties of the structural system in various frequency bands after performing wavelet packet transform (WPT). Therefore, the presence of structural damage can be detected by the wavelet packet energy spectrum.

This paper presents an efficient method to locate the position and evaluate the severity of nut looseness in real-time based on a remote measurement system. The damage location and severity are investigated by the new approach based on the combination of the wavelet packet energy spectrum and statistical theory. An experiment is performed on a high-speed railway experimental platform to test the proposed method.

## 2. Monitoring System Overview

**Figure 2:**
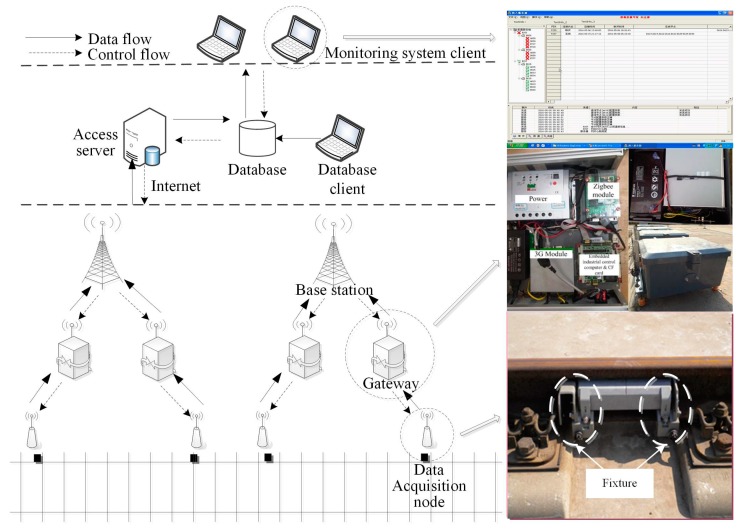
Architecture of the monitoring system.

A remote measurement system is proposed to monitor the state of rail tracks by the Dalian University of Technology [18]. As Figure 2 shows, the system consists of data acquisition nodes, gateways, base stations, an access server, a database client, and a monitoring system client. The data acquisition node collects vibration acceleration data using a uniaxial piezoresistive acceleration sensor which is incorporated into the fixture. The data are uploaded to gateway via a Zigbee network. The base station communicates with gateway using a 3G network. The access server obtains data from the Internet and stores the data into a database. Users can access the database through Web service technology to obtain data. The solar power technology is used to prolong the lifetime of the monitoring system.

**Figure 3:**
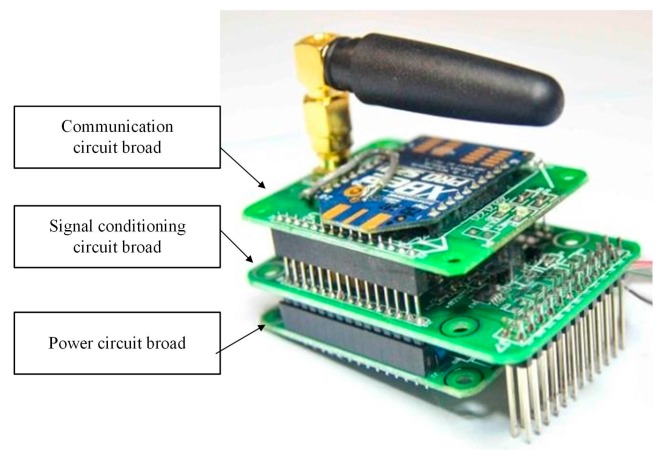
Photograph of the data acquisition node [18].

According to different functions, the data acquisition node (see Figure 3) is composed of a power circuit board, a signal conditioning circuit board, and a wireless communication circuit board. The power circuit board is designed to provide a stable working voltage for the other two boards. The vibration signals with high sampling rate are temporarily stored in an external large-capacity NAND FLASH after conditioning, filtering, and ADC discrete sampling by the signal conditioning circuit board. The data are uploaded to a gateway by a communication circuit board via a Zigbee network after sampling is finished. 

## 3. Materials and Methods

### 3.1. High-Speed Railway Experimental Platform

**Figure 4:**
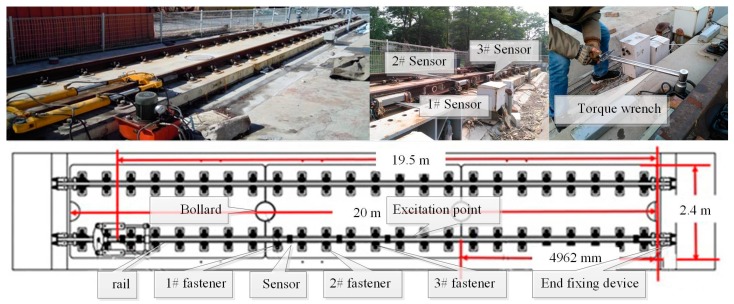
The scene of the experimental platform.

Figure 4 shows the whole scene of the experimental platform including the arrangement of data acquisition nodes and the serial number of fasteners. The uniaxial accelerometer (Model 52M32 accelerometer, Measurement Specialties Inc., Mansfield, TX, USA), based on an advanced piezoresistive MEMS sensing element, was used in the experiment. The railway track was built according to the standard of the Harbin-Dalian high-speed railway. The rails were locked by WJ-7 type fasteners. According to China railway track design specifications, 140 N·m was selected as the theoretical installation value of the nut. Therefore, the severity of fasteners looseness could be defined as the ratio variation of the theoretical installation torque and the actual torque. Three data acquisition nodes were arranged on the spot. The exciting hammer (YD-5T, China Academy of Railway Sciences, China), composed of a plastic hammerhead, force sensor, and handle, was used to excite the rail. The hammer which is widely used to obtain the track parameters [19,20] can provide a stable excitation pulse signal. The sampling frequency was set to 5 kHz.

### 3.2. Wavelet Packet Theory

In wavelet transform, a signal would be decomposed to approximations and details. The approximations are the lower-frequency components of the signal, while the details are the higher-frequency components. Then, the approximation set, in turn, would be decomposed into approximations and details at every decomposition level. In WPT, the details, as well as the approximations, would be split off. WPT is the further development of the wavelet transform, and it can offer better resolution than that of the wavelet transform [21,22,23,24,25]. The acceleration signal *f*(*t*) is decomposed into 2^*j*^ wavelet packet decomposition components when the level of the decomposition is *j*. This can be expressed as:

(1) $$f\left(t\right)=\sum_{i=1}^{2^{j}}f_{j}^{i}\left(t\right)=\sum_{k}c_{j,k}^{i}\psi_{j,k,i}\left(t\right),$$

where *t* is time lag; $c_{j,k}^{i}$ is the WPT coefficient, which can be given by:

(2) $$c_{j,k}^{i}=\int_{-\infty }^{+\infty }x\left(t\right)\psi_{j,k,i}\left(t\right)dt,$$

*i*, *j*, and *k* are three indices denoting the modulation, the scale, and the translation parameter, respectively. $\psi_{j,k,i}\left(t\right)$ is a set of standard orthogonal basis. Moreover, $\psi_{j,k}^{m}\psi_{j,k}^{n}=0$ for *m* ≠ *n*. The WPT component energy can be defined as:

(3) $$E_{j,i}=\int_{-\infty }^{+\infty }{\left[f_{j}^{i}\left(t\right)\right]}^{2}dt.$$

The wavelet packet energy spectrum vector is as Equation (4).

(4) $$V_{E}=\left\{E_{j,i}\right\}=\left[E_{j,1},E_{j,2},\ldots ,E_{j,2^{i}}\right].$$

The total energy can be obtained as follows:

(5) $$E=\sum V_{E}.$$

### 3.3. Choice of Wavelet Basis and the Level’s Number

Wavelet packet transform is a sequence which is composed of a series of wavelet bases. Its essence is the similarity of the wavelet basis and the original signal. The *l^p^* norm entropy was used as the cost function to evaluate whether the chosen wavelet basis and the decomposition level satisfy the requirement or not. The cost function can be defined as:

(6) $$\begin{array}{cc} S_{L}(E_{j})={\sum_{i}\left|E_{j,i}\right|}^{p} & 1\leq p\leq 2. \end{array}$$

**Figure 5:**
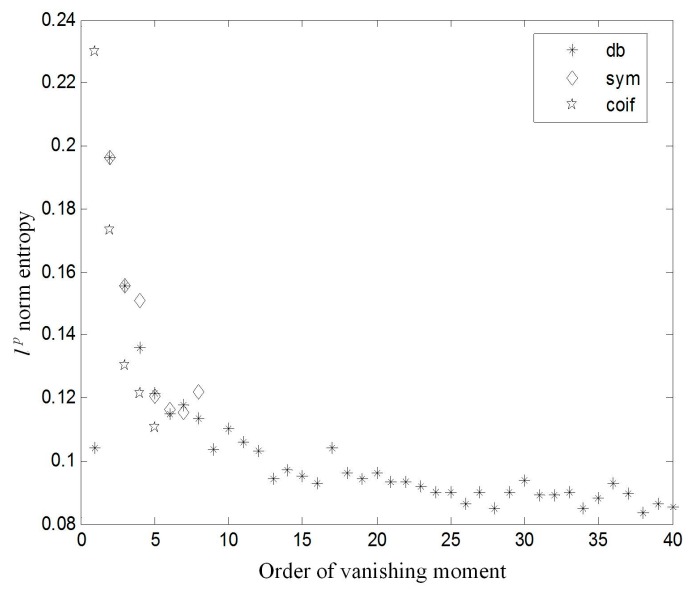
*l^p^* norm entropy of the wavelet function of Daubechies wavelet (dbN), Coiflet wavelet (coifN), and Symlets wavelet (symN).

The smaller the norm entropy is, the more the localized information of the original signal can be represented. The acceleration signal acquired from sensor #3 in a healthy state was selected as an example. The decomposition level was 8. The *l^p^* norm entropy (*p* = 1.5) of Daubechies wavelet (dbN), Coiflet wavelet (coifN), and Symlets wavelet (symN) are shown in Figure 5. It can be found that the cost function of coifN and symN are smaller than dbN when the order of the vanishing moment is less than 8. When the order of vanishing moment is greater than 8, the *l^p^* norm entropy of dbN decreases, along with the increase of the order of the vanishing moment. Therefore, the db40 wavelet is the best choice to process the acceleration signals in this work.

The wavelet packet decomposition level depends on the cost function and the corresponding computation time. The smaller the cost function and the computation time are, the more concentrated the time-frequency energy of the signal and the more time-frequency localized information can be reflected. The decomposition level of acceleration signal was set from 1 to 12, and the corresponding cost function and computation time are recorded in Table 1. It can be found that the corresponding cost function value decreases and the function operation time increases gradually as the decomposition level increases. When the decomposition level is 8, the cost function and the computation time are relatively small. Therefore, the number of decomposition level used in this work is 8.

### 3.4. Wavelet Packet Frequency Band Extraction

The frequency range of the sub-band is limited after the signal transformed by the wavelet packet. It is possible that the frequency of the sub-band is not the main frequency of the original signal. Meanwhile, as the decomposed level increases, the calculation amount of the reconstructed signal increases. The principle of correlation was used to eliminate these useless sub-bands and reduce the amount of calculation. The first m frequency bands were selected to reconstruct the signal. When the reconstructed signal and the original signal correlation coefficient is greater than 0.8, it is considered that the reconstructed signal and original signal has same main frequency components. The acceleration signals of sensor #3 in five operating conditions were decomposed by db40. The number of decomposition level was 8. The correlation coefficients of the reconstructed signals and original signals are shown in Table 2. *m* is the number of selected sub-bands. It can be found from Table 4 that, when the value of *m* is 192, the correlation coefficients are greater than 0.8. Therefore, the former 192 characteristic bands are selected to reconstruct the signal.

### 3.5. Damage Location Index Based on WPT

The change of the signal in the time-frequency domain can be identified by the WPT component energy. The relative energy can be written by the following formula:

(7) $$I_{j,i}=\frac{E_{j,i}}{E}.$$

In order to describe the state of rail fasteners accurately, the energy relative variation (ERV) can be defined as follows:

(8) $$ERV_{i}=\left|\frac{I_{di}-\bar{I_{hi}}}{\bar{I_{hi}}}\right|,$$

where $\bar{I_{hi}}$ is the mean value of the relative energy in the healthy state, *I_di_* is the relative energy under testing condition. The energy accumulation relative variation (EARV) can be defined as Equation (9).

(9) $$\begin{array}{cc} EARV_{k}=\sum_{i=1}^{2^{j}}ERV_{i} & k=1,2,\ldots ,n. \end{array}$$

Suppose that *n* data acquisition nodes are distributed along the rail and *n EARV_k_* values can be obtained. The upper confidence limits of *EARV_k_* at confidence level 1 − *α* can be written as:

(10) $$UL=\mu +Z_{\alpha }\left(\frac{\sigma }{\sqrt{n}}\right),$$

where *μ* and *σ* are the mean and standard deviation of *EARV_k_*, respectively; *Z_α_* is the quantile of the standard normal distribution at the confidence level 1 − *α*; *α* = 0.02. The damage location index (LI) can be defined as:

(11) $$LI=EARV_{k}-UL.$$

When LI is greater than zero, it indicates that the probability of fastener looseness at this position is 100 × (1 − *α*)%. On the contrary, there is no fastener looseness at this position.

### 3.6. Damage Severity Index Based on WPT

The damage severity index (SI) based on WPT is proposed to accurately describe the severity of rail fastener looseness. The wavelet packet energy relative variation deviation (WPERVD) can be defined as follows:

(12) $$WPERVD=\sqrt{\sum_{j=1}^{2^{i}}{\left(ERV_{j}-\bar{ERV_{j}}\right)}^{2}},$$

where $\bar{ERV_{j}}$ is an average value of *ERV_j_*.

The WPERVD can effectively identify the severity of fastener looseness when the excitation remains constant. However, it is difficult to ensure that the excitation is exactly same in spite of using artificial excitation or train excitation. Therefore, the statistical theory is used to calculate the upper confidence limits of WPERVD under different operating conditions:

(13) $$UCL_{\alpha }=\bar{WPERVD}+\frac{\lambda S}{\sqrt{n}},$$

where *UCL_α_* is the upper confidence limit of WPERVD at the 1 − *α* confidence level; *α* = 0.05; $\bar{WPERVD}$ is the average value of *WPERVD*; *S* is the sample standard deviation of WPERVD; *n* is the sample size; and *λ* is quantile which is determined by the t-distribution table. The SI can be defined as follows:

(14) $$SI=\left|UCL_{\alpha }^{h}-UCL_{\alpha }^{l}\right|,$$

where $UCL_{\alpha }^{h}$ and $UCL_{\alpha }^{l}$ are the upper confidence limits of WPERVD under healthy and loose states, respectively.

## 4. Experimental Results

### 4.1. Damage Location Identification

Four operating conditions, including healthy state, one fastener loose (setting of the tightening torque of nut to 0 N·m), two fasteners loose, and three fasteners loose were set for damage location identification (Table 3). Under each operating condition, the rail was excited 30 times at the same point.

**Figure 6:**
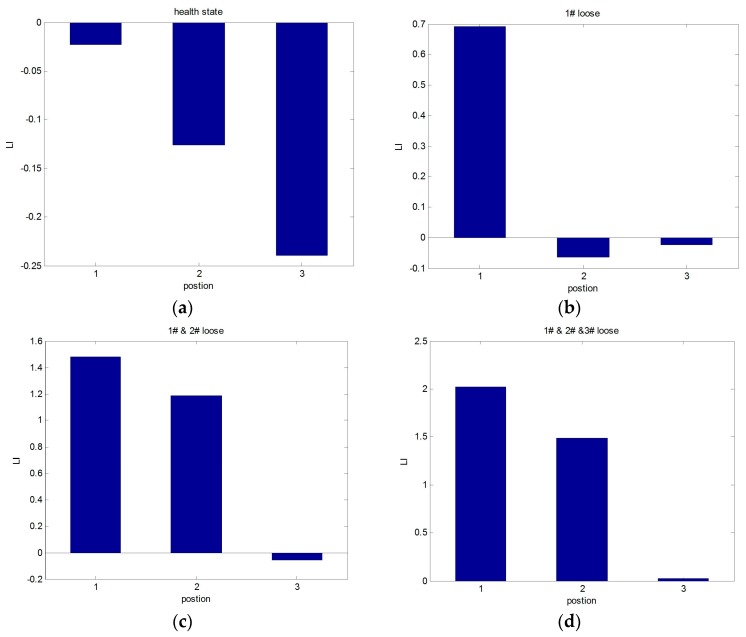
Loose location identification. (**a**) The calculation results of LI in the healthy state; (**b**) the calculation results of LI in the #1 fastener loose condition; (**c**) the calculation results of LI in the #1 and #2 fastener loose condition; and (**d**) the calculation results of LI in the #1 and #2 and #3 fastener loose condition.

The location indicator LI was calculated under different operating conditions (Table 1). The experimental results are shown in Figure 6.

It can be seen that the value of LI is positive at the loose fastener position and that it is negative in the healthy state. The results indicate that the proposed method is effective at locating the position of loosening fasteners.

### 4.2. Damage Severity Identification

Five operating conditions were set for damage severity identification (Table 4). Under a certain temperature, the tightening torque of #3 fastener was set to 140 N·m, 105 N·m, 70 N·m, 35 N·m, and 0 N·m, respectively. Under each operating condition, the rail was excited by 35 times at the same point.

**Figure 7:**
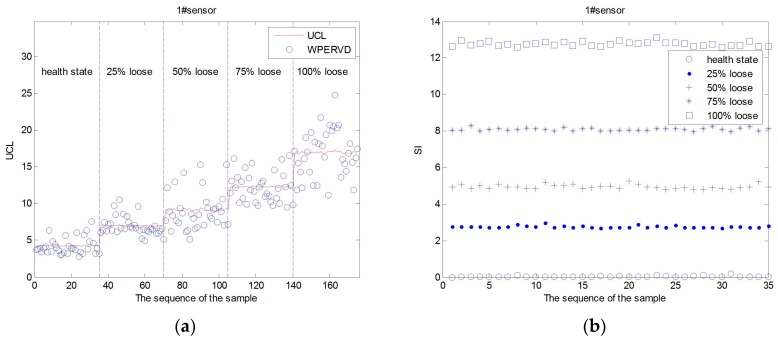
The *UCL*, *WPERVD* and *SI* distribution of sensor #1. (**a**) The *UCL* and *WPERVD* distribution of #1 sensor; and (**b**) the *SI* distribution of sensor #1.

**Figure 8:**
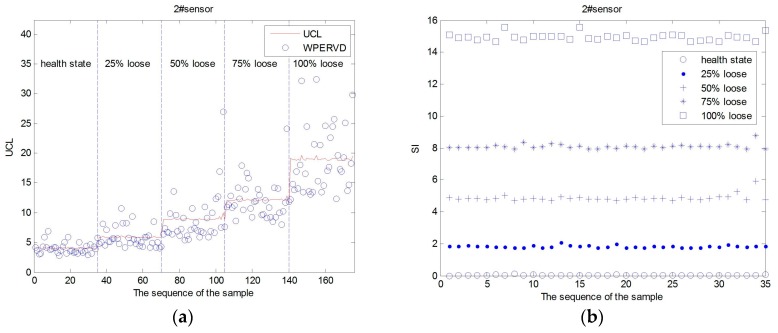
The *UCL*, *WPERVD* and *SI* distribution of sensor #2. (**a**) The *UCL* and *WPERVD* distribution of sensor #2; and (**b**) the *SI* distribution of sensor #2.

**Figure 9:**
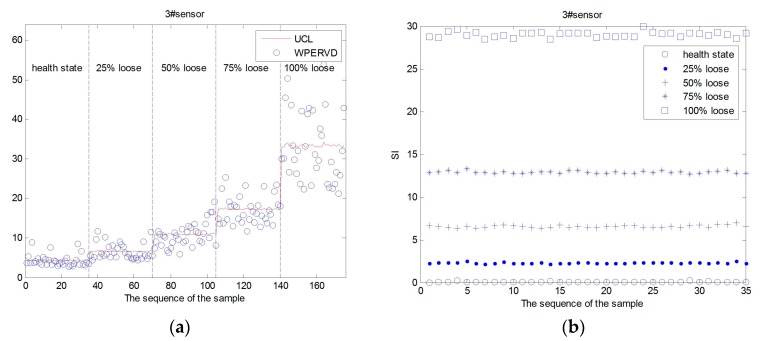
The *UCL*, *WPERVD* and *SI* distribution of sensor #3. (**a**) The *UCL* and *WPERVD* distribution of sensor #3; and (**b**) The *SI* distribution of sensor #3.

**Figure 10:**
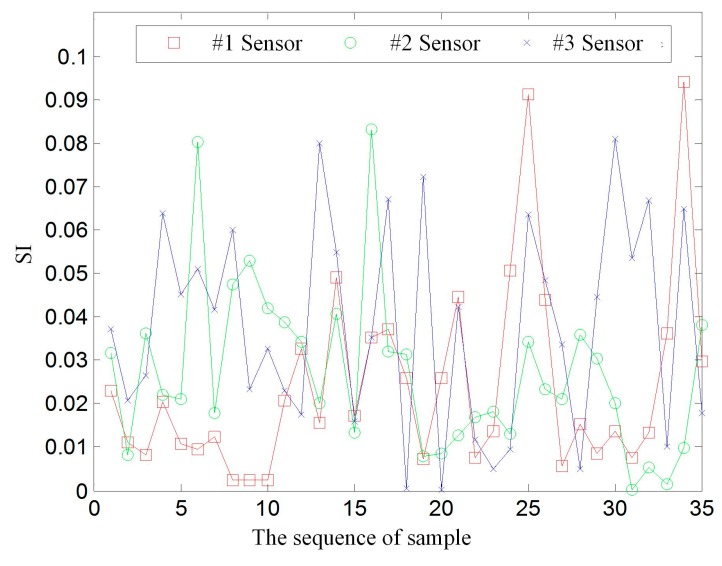
The SI of #1, #2, and #3 sensors of the healthy state.

The WPERVD, UCL, and SI distribution of three sensors under five operating conditions (Table 2) are shown in Figure 7, Figure 8 and Figure 9, respectively. It can be seen that the values of *WPERVD* under damaged states are generally greater than that in healthy states. The results indicate that WPERVD cannot clearly distinguish the condition of rail fasteners, while the value of *UCL* increases with the severity of rail fastener looseness. Therefore, UCL can be used to identify the severity of rail fastener looseness to a certain extent. The values of *SI* under five conditions are obviously different. It can be found that the values of *SI* increases with the severity of rail fastener looseness. Therefore, SI can be used to identify the severity of rail fastener looseness. The values of *SI* in healthy states (Figure 10) fluctuate within ±0.1, which indicates that the proposed method has the advantage of good stability. According to the rail vibration propagation behavior, the sensor far from the excitation location gets less energy. Actually, under the same operating conditions, *SI_3_* > *SI_2_* > *SI_1_*.

According to the experimental results, the relationship between SI and the severity of rail fastener looseness can be described by a polynomial fitting:

(15) *SI*_1_ = −1.028 × 10^−7^*x*^4^ + 3.167 × 10^−5^*x*^3^ − 2.367 × 10^−3^*x*^2^ + 1.502 × 10^−1^*x* + 2.925 × 10^−2^;

(16) *SI*_2_ = 4.573 × 10^−7^*x*^4^ − 7.864 × 10^−5^*x*^3^ + 4.857 × 10^−3^*x*^2^ − 7.575 × 10^−3^*x* + 2.856 × 10^−2^;

(17) *SI*_3_ = 6.604 × 10^−7^*x*^4^ − 8.892 × 10^−5^*x*^3^ + 5.311 × 10^−3^*x*^2^ − 7.074 × 10^−5^*x* + 6.023 × 10^−2^,

where *x* represents the percentage of the looseness degree, *SI*_1_, *SI*_2_, and *SI*_3_ are the severity identification indices of sensor #1, sensor #2, and sensor #3, respectively. An experiment of sensor #1 under four operation conditions was performed to verify the polynomials. According to Equation (15), the corresponding looseness degree can be obtained, and the results are shown in Table 5.

It can be seen from Table 5 that the maximum relative error of the calculated value and truth value is 0.868%. The relative error is within the range of ±1%. Thus, the SI can accurately describe the severity of rail fasteners looseness.

## 5. Conclusions

In this work, a remote measurement system was used to monitor the condition of a rail fastening system. An efficient method was investigated to evaluate the condition of rail fastening system. The damage location and severity of rail fasteners can be identified accurately by LI and SI based on the combination of WPT and statistical theory. The LI can locate the position of loosening fasteners accurately, whether under the conditions of a single fastener loose or several fasteners loose. The value of *SI* increases with the severity of rail fastener looseness. The damage severity of the fastener can be deduced by SI, and the relative error between the calculated value and the truth value is within the range of ±1%.

## Figures and Tables

**Table 1:** The cost function and the computation time of different decomposition levels with the db40 wavelet.

Decomposition Level	Cost Function	Computation Time (s)
1	3.085	0.055
2	1.861	0.056
3	1.492	0.063
4	0.938	0.075
5	0.589	0.102
6	0.307	0.172
7	0.159	0.291
8	0.085	0.600
9	0.046	1.375
10	0.030	3.504
11	0.027	9.892
12	0.018	31.135

**Table 2:** The correlation coefficients between different reconstructed signals and original signals.

Operating Condition	*m* = 128	*m* = 154	*m* = 180	*m* = 192
1	0.732	0.749	0.808	0.877
2	0.740	0.760	0.783	0.886
3	0.718	0.738	0.826	0.864
4	0.718	0.747	0.819	0.871
5	0.712	0.743	0.797	0.871

**Table 3:** Operating conditions of damage location identification.

Serial Number	1	2	3	4
Number of loose fasteners	0	1	2	3
Location of loose fasteners	Null	#1	#1 and #2	#1 and #2 and #3

**Table 4:** Operating conditions of damage severity identification.

Serial Number	1	2	3	4	5
Tightening torque (N·m)	140	105	70	35	0
Damage severity (%)	Null	25	50	75	100

**Table 5:** The test results of sensor #1 under different conditions.

Serial Number	SI	Calculated Looseness Degree (%)	Actual Looseness Degree (%)	Relative Error (%)
1	2.241	24.960	25	0.160
2	2.221	24.816	25	0.736
3	6.431	50.434	50	0.868
4	6.394	50.241	50	0.482
5	13.245	74.835	75	0.220
6	13.211	74.752	75	0.331
7	30.159	99.879	100	0.121
8	30.581	100.285	100	0.285
